# Niche as a Determinant of Word Fate in Online Groups

**DOI:** 10.1371/journal.pone.0019009

**Published:** 2011-05-12

**Authors:** Eduardo G. Altmann, Janet B. Pierrehumbert, Adilson E. Motter

**Affiliations:** 1 Northwestern Institute on Complex Systems, Northwestern University, Evanston, Illinois, United States of America; 2 Departamento de Física, Universidade Federal do Rio Grande do Sul, Porto Alegre, Rio Grande do Sul, Brazil; 3 Max Planck Institute for the Physics of Complex Systems, Dresden, Germany; 4 Department of Linguistics, Northwestern University, Evanston, Illinois, United States of America; 5 Department of Physics and Astronomy, Northwestern University, Evanston, Illinois, United States of America; Universita' del Piemonte Orientale, Italy

## Abstract

Patterns of word use both reflect and influence a myriad of human activities and interactions. Like other entities that are reproduced and evolve, words rise or decline depending upon a complex interplay between their intrinsic properties and the environments in which they function. Using Internet discussion communities as model systems, we define the concept of a *word niche* as the relationship between the word and the characteristic features of the environments in which it is used. We develop a method to quantify two important aspects of the size of the word niche: the range of individuals using the word and the range of topics it is used to discuss. Controlling for word frequency, we show that these aspects of the word niche are strong determinants of changes in word frequency. Previous studies have already indicated that word frequency itself is a correlate of word success at historical time scales. Our analysis of changes in word frequencies over time reveals that the relative sizes of word niches are far more important than word frequencies in the dynamics of the entire vocabulary at shorter time scales, as the language adapts to new concepts and social groupings. We also distinguish endogenous versus exogenous factors as additional contributors to the fates of words, and demonstrate the force of this distinction in the rise of novel words. Our results indicate that short-term nonstationarity in word statistics is strongly driven by individual proclivities, including inclinations to provide novel information and to project a distinctive social identity.

## Introduction

Much information about the fabric of modern human society has been gleaned from large-scale records of human communications activities, such as time stamps and network structures for email exchanges, mobile phone calls, and Internet activity [1]–[4]. But the flow of words has the potential to be even more informative. Words characterize both external events and otherwise unobservable mental states. They tap into the variety of experience, knowledge, and goals of different interacting individuals. The word stream is information-dense, because the number of distinct words and expressions is so great. The lexicon of a literate adult is estimated to contain over 100,000 distinct items [5], and it continues to grow as new words are encountered [6].

Records of the linguistic transactions within a community provide an ongoing statistical sampling of the vocabulary of a language. The sample at any time reflects both the social context (who is speaking, and to whom) and the topical context (what they are speaking about). But the language dynamics does not just passively mirror the context. Language adapts to new circumstances and needs through lexical innovation [7]. Large datasets available from the Internet provide an unprecedented opportunity to study the dynamics of words, as well as phrases and tags [8]–[11]. Here, we explore lexical fluctuations in relation to both individuals and topics by analyzing records of Usenet groups. Created over one decade before the World Wide Web, the Usenet groups were amongst the first systems for world-wide exchange of messages on the Internet. Usenet archives reveal the rise of “Netspeak”, the language nowadays widely used on the Internet and in telephone text messages [12]. The groups we studied, rec.music.hip-hop and comp.os.linux.misc, were selected for their great lexical creativity. In these datasets, users serve as proxies for individuals, and threads as proxies for topics (see *Methods*). Our study goes beyond the analysis of user activity in Usenet groups [13], and focuses instead on the content of the messages.

It is known that word frequency is a factor in frequency dynamics on historical time scales [14], [15], a finding that is expected from models of language learning across human generations [16]. Here, we identify two new factors–-the dissemination of words across individuals (users) and the dissemination of words across topics (threads)–-and we develop a method to quantify dissemination that controls for word frequency. Because words are acquired and reproduced by users as they communicate with each other about different topics, these two dissemination measures serve to characterize two important dimensions of the word niche. We apply these measures to demonstrate that dissemination is a much more powerful determinant of word fate than word frequency is; poorly disseminated words are more likely to experience a frequency reduction than widely disseminated words.

These results suggest analogies between word fates and the fates of biological species. In population biology, the term *niche* refers to the relationship between a species and the aspects of its environment that enable it to live and reproduce. Quantifying the breadth and versatility of a species' niche, as distinct from the species' sheer abundance, is key to understanding its competitive position within an ecosystem [17]. The geographic size of the niche is a statistical correlate of species duration, as species with large ranges are less likely to become extinct [18], [19]. Analogies between language and population biology have proved fruitful in understanding the dynamics of entire languages, in particular the relationship of community size to overall rates of linguistic change [20], [21] and to properties of the syntactic and morphological systems [22]. Here, we work at a more fine-grained level, quantifying the impact at short (two-year) time scales of the heterogeneous usage of language inside a community. Because we consider the role of heterogeneity amongst people within the community, the results also support comparisons between the dynamics of the linguistic system and other social dynamics, such as the spread of opinions or the popularity of news items, videos, and music [23], [24].

The relation with social dynamics is strengthened by a case study of novel words with rising frequency, in which we compare a set of words for products and public figures to a set of slang words. The rise in use of words in the first set is mainly driven exogenously by events that are external to the Usenet group, such as product releases, political crises, and public performances. Because the use of slang words is strongly influenced by the social values and patterns of communication within any given linguistic group [25], [26], the use of the (slang) words in the second set should be more influenced by factors endogenous to the Usenet community. The force of this distinction in word dynamics mirrors its force in other social behaviors, ranging from YouTube viewing to scientific discoveries, marketing successes, financial crashes, and civil wars [27], [28]. Finally, we explore the correlations between individuals and topics as dimensions of word dissemination. The two dimensions are shown to be separable, and individual choices prove to be more important than topic in determining patterns of word usage. These results highlight the importance of individuality in the use of language, and imply limits on the role of social influence and social conformity.

## Results

### Dissemination of words across users and threads

If everyone knew the same words, and chose to use them at random with their given frequencies, the dissemination of words across users would be the result of a Poisson process. We are interested in the extent to which the actual number of users of each specific word deviates from this baseline model. We define the measure of dissemination of each word 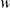 across users as
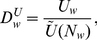
(1)where 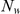 is the number of occurrences of the word in the dataset, 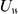 is the actual number of users whose posts include word 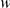 at least once, and 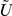 is the expected number of users predicted by the baseline model. The latter is determined from 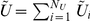, where 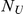 is the number of users and 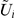 is the probability that user 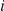 used 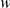 at least once when all the words in the text are shuffled randomly (see *Methods*). Dissemination across threads is analogously defined as
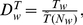
(2)where 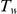 is the number of threads in which the word appears, and 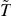 is the corresponding expected value from the baseline model. The word frequency is defined as 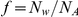, where 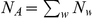 is the total number of words in the dataset; 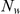 is a count, and the frequency 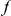 normalizes this count to a probability. In the rest of the paper, we focus on the properties of the dissemination measures 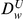 and 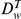, or 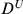 and 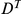 for notational simplicity.

**Figure 1:**
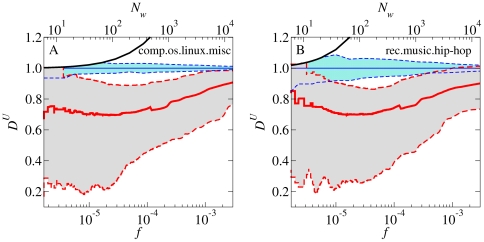
Relationship of frequency 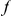 to dissemination across users 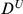. **A**, **B**, The results are shown for half-year windows centered on 1998-01-01 for the comp.os.linux.misc group (**A**) and the rec.music.hip-hop group (**B**). Red solid line: running median for all words with 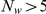. Red dashed lines: 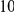
th and 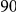
th percentiles for the same words. Blue dashed lines: 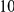
th and 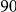
th percentiles around the expected value of 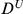 for randomly distributed words, determined by Monte Carlo simulations with 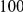 independent shufflings of the text. Black line: analytically calculated ceiling 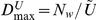 (floor effects and the other ceiling, 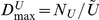, do not pertain within the scale of the figure). The median empirical 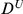 is systematically below the 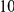
th percentile of the estimated random variation. The relationship of median 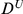 to 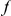 is nearly flat up to 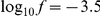.

The expected value of 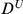 is 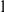 for a word of any frequency that is distributed randomly across users. 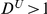 indicates *over-disseminated* words and 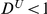 indicates concentrated or *clumped* words. For example, in a half-year window centered on 1998-01-01 in the comp.os.linux.misc group, the words *thanks* and *redhat* have almost identical frequencies, but contrast in their dissemination (*thanks*: 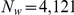, 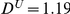; *redhat*: 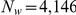, 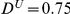). A similar contrast is provided for the same time window in the rec.music.hip-hop group by the words *please* (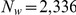, 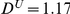) and *article* (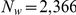, 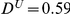). The measure 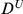 exhibits a lower bound determined by the number of occurrences of the word: 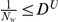. For any given set of posts, there is also an upper bound determined by the relationship of 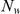 and 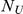 to 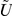: 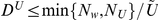. Due to the discreteness close to the lower bound, we set a threshold 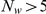 for the computation of 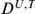. The few dozen most frequent words (mainly common function words) are also omitted from our analysis, because 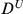 is not informative when 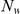 is too large compared to the number of users. Figure 1 shows results on the expected statistical fluctuation around 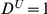 for randomly distributed words in a representative window of each Usenet group, as determined by a Monte Carlo simulation. The upper and lower extremes of the fluctuation depend on frequency, but only slightly.

The dissemination across threads 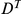 is closely related to the *residual inverse document frequency* (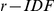), a measure used in text processing to characterize the extent to which a word is associated with particular documents [29], [30]. 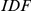, defined as the reciprocal of the number of documents in which the word occurs, is strongly influenced by word frequency. *Residual* 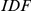 addresses this artifact by taking the difference 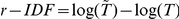, where 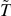 is approximated using a Poisson baseline model with equal document lengths. When this condition holds, 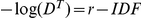. The measure 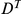 is a generalization of 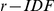 that remains valid when the lengths of the documents are very unequal, as for the present datasets (see Figure S1 in Supporting Information S1).

### 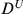 and 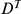 as predictors of word fate

**Figure 2:**
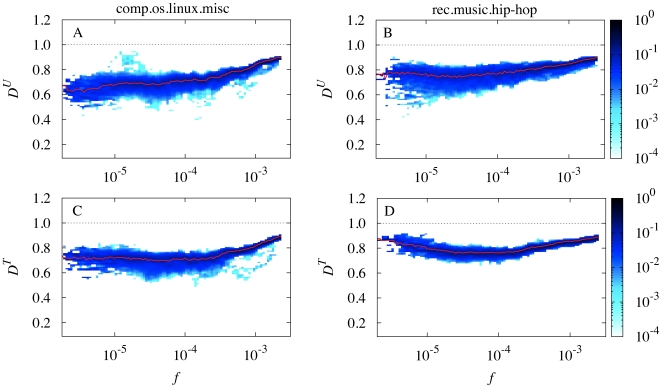
Summary of the relation between frequency 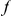 and dissemination across users 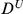 and threads 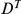. The running median shown in Figure 1 is now calculated in all half-year windows. **A–D**, Results for both the comp.os.linux.misc group (**A**, **C**) and the rec.music.hip-hop group (**B**, **D**). The color code indicates densities in the range of 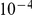 (light blue) to 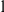 (dark blue) obtained by combining all running medians, while the red line indicates the median of the resulting, combined distribution.

To explore the changes over time in the statistical attributes of words, we begin by partitioning each dataset into non-overlapping half-year windows. Figure 1 displays the behavior of 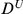 within a representative half-year window for both groups. Most words are significantly clumped. At all word frequencies, the median 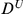 falls below the 10th percentile for random fluctuation of the expected value under the baseline model. For words with 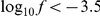, 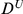 varies considerably and is not correlated with frequency 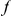. Words with 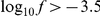 are extremely high-frequency words, and comprise less than 0.5% of all distinct words in this window. But even these words are somewhat clumped. These findings are reproduced in all half-year windows for both Usenet groups, as summarized in Figure 2AB. They provide the user counterpart to prior observations of clustering of words in documents and in time [8], [29]–[32].

**Figure 3:**
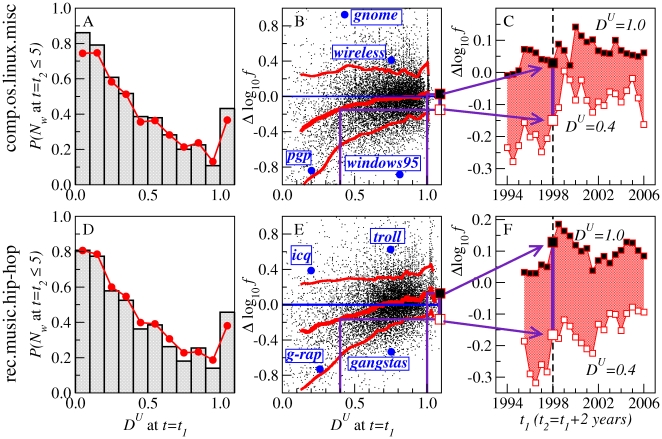
Dissemination across users 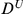 as a predictor of falling below threshold and of frequency decay. The analysis is performed over half-year window pairs 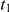 and 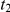 separated by two years for the comp.os.linux.misc and rec.music.hip-hop groups. **A**, **D**, Fraction of words with 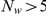 in 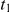 that fall to 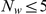 in 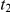. Histogram in gray: results from selected window pairs centered on 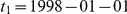 and 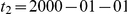. Red line: average over different non-overlapping window pairs with 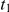 ranging from the (rounded off) beginning of the group through 2006-01-01, and 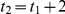 years. The probability of falling below threshold goes down as 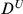 increases. **B**, **E**, Scatter plots of all words with 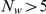 in both windows (12,883 words for comp.os.linux.misc, 12,237 words for rec.music.hip-hop). Values on 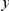-axis: log-frequency change 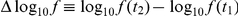. Red lines: running median, 10th percentile, and 90th percentile. Words with rising frequency appear above and words with falling frequency appear below 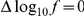. Examples of words with large frequency changes are highlighted. The probability of frequency decay is greater for words with low 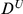. **C**, **F**, Summary of the dominant pattern in panels **B**, **E** over all non-overlapping windows with 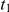 ranging from the beginning of the group to 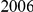, and 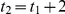. Median values of 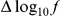 at 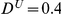 and 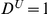 are shown for each pair of windows.

We now examine 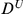 as a predictor of frequency change for words over two-year periods. We first note that 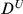 is strongly related to the likelihood that a word with 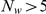 in a window 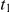 falls below this threshold in a window 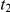 taken two years later. This is illustrated for both Usenet groups in Figure 3AD, where 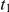 and 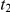 mark the centers of the half-year windows. The finding is so statistically robust that it is reproduced for every choice 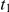 and 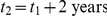, in both groups. The same pattern is also mirrored in the frequency changes of words that are above the 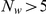 threshold at both 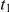 and 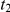. Within this group of words in the selected window of comp.os.linux.misc, 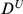 is a strong predictor of whether the word rose or fell in frequency (Figure 3B). In the selected window of rec.music.hip-hop, 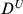 is likewise a strong predictor of the changes in word frequencies (Figure 3E). The consistency of this pattern over all windows may be seen by comparing 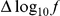 for words with 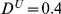 and with 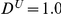, values that span the well-populated portion of the range in 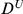. Words with the former value tend to decline in frequency (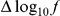 is negative), while words with the latter value tend to maintain or increase their frequencies (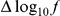 is near zero or positive). There is no 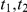 pair for either dataset in which the effect is reversed (Figure 3CF).

**Figure 4:**
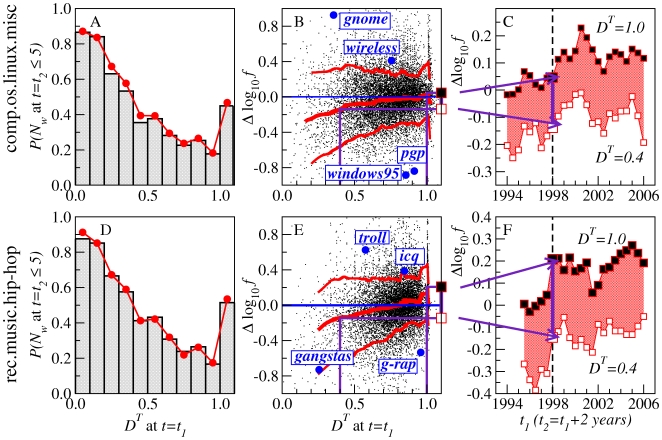
Dissemination across threads 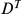 as a predictor of falling below threshold and of frequency decay. This figure is the 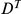-counterpart of Figure 3.

**Figure 5:**
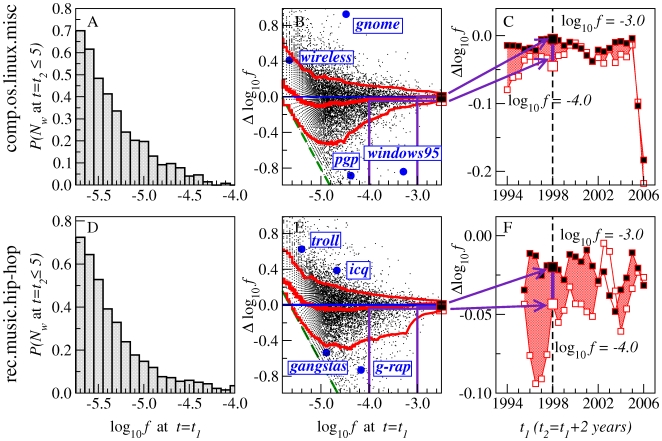
Frequency 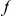 as a predictor of falling below threshold and of frequency decay. This figure is the 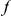-counterpart of Figure 3. The dashed green lines in panels **B**, **E** indicate the minimum possible 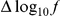 for a given 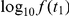, due to the threshold 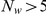 imposed at 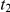. The analysis in Table 1 includes only the range 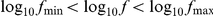, where 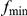 and 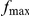 are the limits of the range considered. The range is truncated at 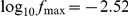 because, for words above this frequency, 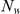 is so large compared to the number of users or threads that 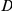 is not informative. The range is truncated at 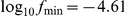 for comp.os.linux.misc (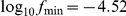 for rec.music.hip-hop) because below these cutoffs the exclusion of words falling under the threshold (i.e., 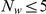) introduces artifacts in the relationship to 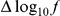 (c.f. the relationship of the dashed green lines to the 10th percentile line). Specifically, 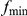 was chosen for each dataset so that the percentage of words falling below the threshold at 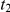 would be less than 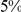 of the words with 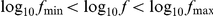.

This far, our analysis has focused on 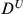. In sociolinguistic parlance, we have considered the “indexicality” of words, that is the extent to which words are associated with individuals or types of people. Now, let us also consider 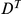, our measure of “topicality” (dissemination across topics). As shown in Figure 2CD and in Figure 4, the results just described for 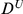 also hold for 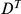. The connection between 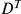 and frequency change agrees with Ref. [33]'s study of foreign borrowings in news articles. What is the relative importance of these factors in predicting frequency change? As Table 1 shows, 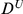 is more important than 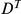. Moreover, both are more important than 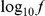, whose importance is comparatively slight, as shown in Figure 5.

**Table 1:** Relative importance of dissemination across users, dissemination across threads, and frequency in word dynamics.

Group	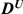	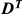	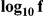
comp.os.linux.misc	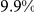	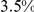	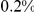
rec.music.hip-hop	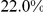	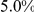	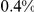

Relative importance of the three factors as predictors of frequency change (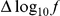), calculated using the method of Ref. [62]. Importance is based on the fraction of the variance of 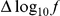 explained by each factor. This method conservatively estimates the relative importance of the independent variables in a multiple regression setting. The data are combined over all window pairs 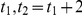 considered in Figure 3. To avoid artifactual correlations for small and large 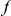, the range of words is restricted in 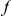, as indicated in the caption of Figure 5.

Words change over time not just in their frequency, but also in their dissemination. A signal aspect of changes in 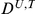 is a strong negative correlation with frequency change (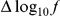). For comp.os.linux.misc, the correlations of 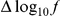 with 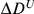 and 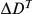 are 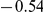 and 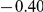, respectively; for rec.music.hip-hop, 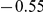 and 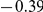, respectively. These negative correlations can be understood by comparing two scenarios. In one scenario, a word rises in frequency because it becomes more widely used; it is used by more individuals and/or in the discussion of more topics. In this scenario, the increase in frequency is accompanied by steady or increasing values of the dissemination measures 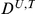. In a contrasting scenario, a word rises in frequency without a concomitant increase in the number of users and/or topics, because it is used more repetitively by the same few people and/or in discussing the same topics. In this scenario, the increase in frequency is accompanied by decreasing values of 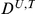, because the use of the word becomes more and more concentrated in comparison to what the random baseline would predict. In this case, it follows from Figure 3 that the resulting low 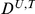 puts the word at risk of declining in frequency thereafter. Just as a population that explodes in a narrow ecological niche may well crash later, it appears that repetitive communications are more discounted than emulated by others. This picture broadly resembles recent observations about buzzwords in the blogosphere, which are reported in Ref. [11] to exhibit great fluctuations in their frequencies, as well as an apparent association between a fast rise and subsequent obsolescence. The fact that the correlations of frequency change (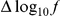) with dissemination change (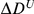 and 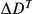) are strongly negative means that the second scenario is the dominant one in our datasets. Overall, fluctuations in frequency driven by variability in user behavior and topic dominate the statistical behavior, with the result that patterns similar to those in Figures 3 and 4 are also observed by making the same calculations in the reversed time direction (that is, by relating 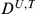 at 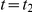 to 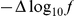). These large, short-term fluctuations add an important new dimension to the study of the long-term dynamics of language, as any novel expression must survive in the short term to survive in the long term.

### Case study: Rising slang and product words

A new word must establish itself in a niche to survive in the language. The survival rate of lexical innovations is not known, but any successful innovation must have overcome short-term fluctuations in 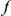 that risked driving it to an early extinction. We now present a case study of successful innovations. First we identify all words that were not used during the first years of the group, and that were consistently used for at least some years thereafter (for precise thresholds, see Text S2 in Supporting Information S1). From this collection of rising words, we selected two sets of words for each group. The first set is designated as P-words because they refer to products (such as *gnome*, a desktop environment introduced in 1998) and public figures (such as *eminem*, a rapper popular from the late 1990's). Exogenous factors contribute strongly to their use. The second set, designated as S-words, exemplifies slang words and other novel vernacular language. These novel words were selected with the aid of on-line dictionaries of Internet and Usenet terms (see Text S2 in Supporting Information S1). We consider the dynamics of these words to be more dominated by factors endogenous to the linguistic systems and social networks of the Usenet groups. Although many of the S-words may have been learned from people outside of a Usenet group, such as celebrities seen on television, the group itself is the locus of the the social values and conventions that lead to some celebrities being imitated and others ignored. Paired lists of P-words and S-words were frequency matched to the extent possible. The words and their statistics are listed in Tables S1, S2, S3, S4 in Supporting Information S1.

**Figure 6:**
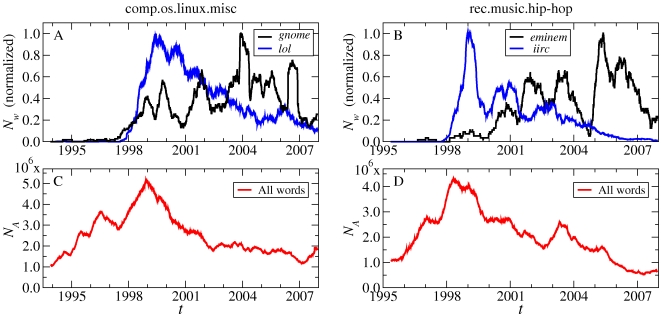
Dynamical behavior of P- and S-words in time. **A**, **B**, Number of occurrences of example P- and S-words as a function of the center 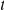 of each half-year window. Example words: P-word *gnome*, a software product; S-word *lol* (“laughing out loud”); P-word *eminem*, a rapper; S-word *iirc* (“if I recall correctly”). The curves are normalized by the maximum number of occurrences per window reached over all windows: 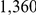 for *gnome* and 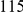 for *lol* (**A**); 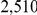 for *eminem* and 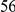 for *iirc* (**B**). **C**, **D**, Total number 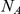 of all words in each half-year window centered at 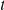.

**Figure 7:**
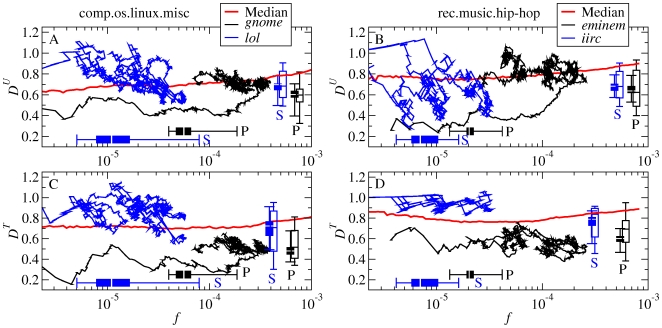
Dynamical behavior of P- and S-words in frequency and dissemination. **A**, **B**, Relationship of 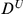 to frequency. Black and blue curves: evolution of example P-words and S-words over time. Red line: median over all words, as in Figure 2. Boxplots: distribution of the mean frequency 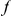 (solid, horizontal), mean dissemination 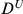 (solid, vertical), and mean dissemination 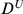 in the rising period (open, vertical) for all P- and S-words (Tables S1, S2, S3, S4 in Supporting Information S1). The mean is calculated over all words with 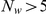 within the corresponding window. **C**, **D**, The 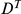-counterpart of panels **A**, **B**.

Figure 6 compares the dynamics of example P-words and S-words. Temporal fluctuations in the total activity of the group (Figure 6CD) provide a backdrop for considering the different fluctuations in the number of occurrences of some typical P-words and S-words (Figure 6AB). Our Usenet database also allows us to go beyond the frequency dynamics of words over time, as explored in Ref. [34]'s recent study of words in books, and look at the roles of topics and individuals in determining this dynamics. In Figure 7, we show the behavior of the words in a frequency-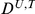 space. As indicated by the horizontal boxplots, the P-words and S-words are located in the frequency region below 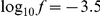, in which the frequency is not correlated with 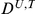. Trajectories over time for two example words are superimposed, beginning when the words first reach 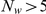. In contrast to the example S-words, the example P-words begin with very low 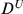 values, and rise greatly in frequency before becoming widely disseminated. The vertical boxplots show that P-words have overall lower 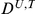 than S-words (though both fall below the median of all words). The contrast in 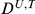 over the entire period is replicated if we consider just the early rising period of each of the words in both groups (see the aggregated statistics displayed in Figure 7, and further details in Tables S1, S2, S3, S4 in Supporting Information S1).

Significant clumping in 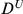 is expected for S-words, because choices of vernacular language such as *lol* (*laughing out loud*) and *prolly* (*probably*) reflect the individual's construction of social identity [35], [36]. How can we construe the finding that P-words are even more clumped in 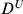 than the S-words are? Recalling that all of the words in the case study were preselected to exemplify rising trends, it seems possible that the highly clumped P-words reflect the distinctive information access of their users. For example, *gnome*, which has a 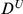 value of 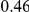 in its early rising period, refers to a graphical desktop environment that was originally created by two Mexican programmers, Miguel de Icaza and Federico Mena. By discussing their experience with this interface, its early adopters bring information to the comp.os.linux.misc group that other users do not yet have. In short, by contributing posts about experiences and activities external to the Usenet group, a small number of users can be the vehicle for exogenous factors to come to influence the vocabulary of the group more generally.

The low 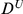 of the P-words and S-words would tend to predict a decline in frequency (see above), but instead the frequencies of these particular words rose. For P-words, the rise is driven by events external to the Usenet community. For example, the P-word *ssh* (from comp.os.linux.misc) refers to the secure shell network protocol. The invention of ssh allowed people to carry out remote file transfers without compromising sensitive information such as passwords. The immediate adoption of this technological improvement is clearly one reason for the rise in use of the word *ssh*. In rec.music.hip-hop, the use of the P-words *bush*, *saddam*, *and iraq* reflects discussion about the war in Iraq. Both the war, and the political events leading up to it, took place outside of the Usenet community. In Figure 6B, the 2005 rise in the frequency of *eminem* reflects heavy media coverage of his possible retirement. The use of the P-words also reflects endogenous factors to some extent. The fact that *bush*, *saddam*, *and iraq* met the inclusion criteria in rec.music.hip-hop, but not in comp.os.linux.misc, suggests that a shared interest in politics is more important within the Usenet hip-hop community than in the Usenet linux community.

However, for the S-words, we consider that the endogenous factors were even more important. For these words, there are alternative ways of referring to the same general concept. In both groups, *lol* competes with *rofl* (rolling on the floor laughing), *ha-ha*, and other expressions. In rec.music.hip-hop, *addy* competes with *address*. In comp.os.linux.misc, *y2k* competes with *year 2000*, and *boxen* (as a plural of *box*, generalizing the jocular plural of *Vaxen* for the *Vax* brand of computers) competes with *boxes*, *servers*, *computers*, etc. The choice of one such word over an alternative expression with the same referent reflects the social value associated with the word, which is a non-referential component of its meaning. By their nature, slang words stand out from other words through being used to “establish or reinforce social identity or cohesiveness within a group, or with a trend or fashion in society at large” [25]. In African-American Vernacular English (the original language of hip-hop), the transitory slang expressions of various subgroups of speakers, such as teenagers and musicians, serves to differentiate them within a larger African-American community sharing a rather stable lexicon and grammar [26]. Reference [12] suggests that on-line groups are especially likely to use jargon and slang as a means of constructing and affirming group solidarity, since the group has no identity outside of its on-line communications. But the use of some S-words also reflects exogenous factors to some extent, which may help explain their success despite the relatively low dissemination. The invention of cell-phone texting probably contributed to the availability of acronyms as slang expressions, the rise of server farms probably contributed to the need for a way to refer to computers as fungible units, and the linguistic influence of a particular rapper might have increased after a successful performance. However, these factors seem weaker than for the P-words, because they do not appear to dictate the particular choice of word out of all the alternatives. Related cases of social dynamics for which a combination of exogenous and endogenous factors has been considered include music downloads [23] and popularity patterns for YouTube videos and for stories on the news portal Digg [37], [38].

By having the lowest overall distribution of 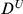 values, the P-words contrast with all other rising words, including both the S-words and typical words whose frequencies increased (as exemplified in Figure 3BE by data points in the upper-right quadrant of each panel). This suggests that exogenous forcing is more efficient than other kinds of forcing. The fact that S-words had higher 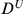 values overall than the P-words did, with no S-word rising from as low a 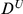 value as the lowest P-words, makes the S-words appear more similar to words in general. In the absence of strong forcing by external events, the social dynamics within the group dominates the word dynamics, with reinforcement by peers providing a natural mechanism for the words to rise. The results support our understanding of 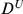 as a determinant of frequency change; high 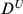 values provide an index of the fact that relatively many different users provide examples of use of a specific word that others may imitate. The 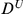 values for S-words are somewhat low compared to the distribution for all words. We can speculate about the mechanisms for this outcome. Exogenous factors in the use of S-words, mentioned just above, may play a greater role than is typical for words in general. Moreover, the force and emotions associated with the social value of the S-words may provide an additional factor driving the dynamics.

Most of our principal observations about the dissemination across users (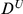) of P-words and S-words are also true for the dissemination of the same words across topics (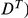, as shown by comparing Figure 7AB to Figure 7CD. Given that the measures 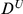 and 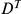 both quantify the relative extent of the word niche, these detailed parallels in the behavior of the two measures raise the question of how many dimensions we are really dealing with. Since people form social groupings around shared interests [39], [40], and choose words that express solidarity with these same groupings, do the two dimensions of indexicality and topicality reduce to just one underlying dimension? Or are the two dimensions separable, even if related through complex interactions? We take up these questions rigorously in the next section.

### Factoring the relative contributions of individuals and topics

We have shown that most words, including both highly indexical words such as slang words and highly topical words such as products, are significantly concentrated in both 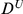 and 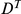. We have sketched some reasons for these dimensions to be positively correlated. How can we rigorously evaluate their separability and relative importance? To address this issue, we consider new measures that effectively factor indexicality and topicality as contributors to 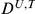, and we standardize the datasets to eliminate distributional artifacts.

We first introduce 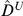 as a modification of 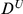 in which 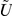 in Eq. (1) is calculated from a baseline model that shuffles the words only within threads, rather than across all users and all threads. Analogously, we introduce 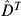 as a modification of 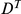 in which 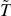 in Eq. (2) is calculated from a baseline model that shuffles the words only within posts of the same user. These new quantities provide a direct measure of the extent to which individuals and topics contribute to the concentration of words observed above. While 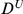 reveals whether the word is clumped or over-disseminated by comparing the actual dissemination with that obtained by “erasing” all the structure, 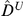 maintains the structure of the threads and considers randomization of words across users within them. If 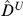 is significantly closer to 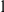 than 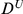 is, then topics must strongly influence the individuals' choice of words. Analogously, the role of individuals can be confirmed by comparing the extent to which 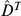 is closer to 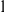 than 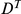 is.

To ensure that users and threads serve as comparable proxies of individuals and topics, we randomly trim the datasets to eliminate the differences in their distributions that are visible in Figure S1 in Supporting Information S1. For each window, the trimming scheme standardizes the user contribution per thread and the size of all posts, matches the number of users and threads, and approximately matches the distribution of posts per user and per thread (see Text S3 and Figure S2 in Supporting Information S1). The trimmed comp.os.linux.misc (rec.music.hip-hop) dataset remains large enough for our statistical analysis, with an average of 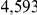 (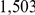) posts and 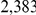 (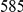) users and threads per half-year window, and an overall average of 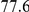 (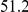) words per post.

The exact distributions of values of 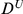 and 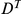 change with the trimming. Trimming generally increases 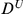 and 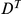 for the words that survive, but the trends and all conclusions from previous sections still stand. For example, the overall median 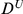 changes from 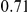 to 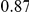, and the overall median 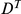 changes from 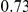 to 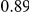, for the comp.os.linux.misc group. The relative differences in both groups remain essentially unchanged, which means that the measures 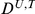 provide meaningful comparisons even when the distributions are not streamlined. However, the trimmed set offers the advantage of providing exact and non-artifactual information about the correlations between the measures.

Table 2 displays the important correlations amongst the original and modified measures. The correlation between 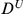 and 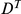 is positive, confirming the expectation that indexicality and topicality are related. But it is far less than 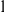, suggesting that 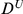 and 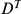 contribute substantially different information. The measures 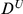 and 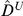, as well as 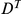 and 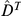 are positively correlated, as expected because these are related measures by definition. Finally, the negative correlation between 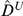 and 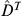 is a confirmation that these quantities partially factor 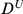 and 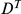 and hence provide the information they are designed to provide. Notice that this negative correlation is possible, despite the positive correlation of the other pairs of variables, because the positive correlations are not all close to one.

**Table 2:** Correlations between dissemination measures.

Group	( 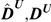 )	( 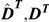 )	( 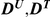 )	( 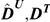 )
comp.os.linux.misc	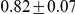	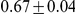	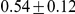	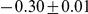
rec.music.hip-hop	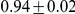	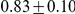	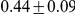	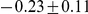

To obtain the correlations, first we calculate 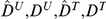 for all words with 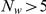 in the half-year windows of the trimmed datasets. The Pearson correlation coefficient, for each pair of variables, is then calculated over all words. The values reported in the table correspond to the averages 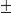 standard deviations calculated over all non-overlapping half-year windows.

**Figure 8:**
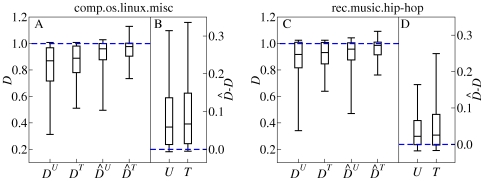
Summary statistics of the dissemination measures. **A**, **C**, The box-and-whisker plots indicate the median, the quartiles, and the octiles for 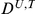 and 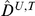 over the collection of all non-overlapping windows of the trimmed datasets. **B**, **D**, Corresponding statistics for 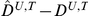 estimated from individual words. The statistics includes all words with 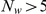 within the corresponding windows, with occurrences in different windows being counted independently.

We now use the trimmed datasets and modified measures to further test the relative importance of indexicality and topicality. As shown in Figure 8AC, 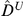 and 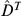 are statistically larger than 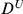 and 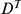, respectively, but they remain smaller than 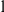. This confirms that most words are clumped with respect to both users and threads. Overall, 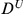 is smaller than 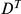, indicating that words are generally more concentrated with respect to users than to threads. This observation is rigorously confirmed by the fact that 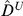 is smaller than 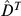 to a comparable extent as 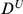 is smaller than 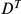. Figure 8BD shows that also for individual words, 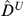 and 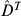 are typically larger than 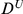 and 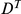, respectively. Furthermore, we can elucidate the effect of threads on users by considering the magnitude of the difference 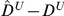, and similarly, the effect of users on threads by considering 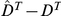. These comparisons reveal that the effect of threads on users is statistically smaller than the effect of users on threads, both in the aggregate (Figure 8AC) and for individual words (Figure 8BD).

The most striking effect shown in Figure 8AC is the large number of words with small 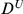 in comparison to 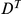. After trimming, over all windows, the comp.os.linux.misc (rec.music.hip-hop) dataset has 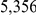 (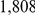) words with 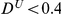, versus 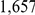 (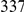) words with 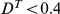. The list of words with 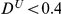 but 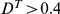 includes both very common words and highly topical words. In comp.os.linux.misc, example words include *imagination*, *coffee*, *angst-ridden*, and *saukrates* (a rapper); in rec.music.hip-hop, examples include *regards*, *baptized* and *tauri* (a Hungarian Warcraft server). It is interesting that such words are even more distinctive to individuals than to topics. A contributing factor to this clumpiness is the use of formulaic expressions. Such expressions, which are found in signature blocks, as well as in other conventionalized communications like greetings and insults, often have quite idiosyncratic lexical choices.

Altogether, we have strong evidence that the lexical make-up of the threads is strongly determined by the individual users. This speaks against the possibility that the topic dictates the vocabulary, and equally against the possibility that mutual imitation causes strong convergence in lexical choices as people interact in the discussion. This is a striking result. It contrasts with the major thrust of research on modeling the evolution of lexical systems, which is to explain convergence in the community [41], [42]. This suggests that individuals may be more autonomous in their choices of words than in a wide range of other behaviors, from yawning and gait [43] to complex conscious decisions like the decision to purchase a product or to vote [44]. Given that individuals use different words to talk about the same topic, that word concentration over users is more extreme than over threads, and that 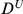 is the strongest predictor of frequency change, the heterogeneity of people emerges as the single strongest factor in lexical diversity, both at any particular time and over time.

## Discussion

We have introduced two new quantities, 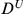 and 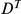, as measures of the dissemination of words across individuals and topics, and used them to characterize the vocabulary of two online discussion groups over a period of more than a decade. We found that almost all words are concentrated with respect to both individuals and topics, and that at short-term (two-year) time scales, the word's concentration in the space of users and topics, as revealed by 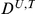, is a strong determinant of word fate. 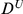 and 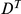 are separable components, and both trump word frequency. However, 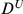 trumps 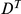.

Word frequencies over time reflect a replicator dynamic, that is, a dynamic in which the words are reproduced by being copied through imitation [20], [41], [42], [45]. Including both learning and use, this dynamic reflects an interaction of social and cognitive factors [46]. Word learning is facilitated by variety in the context of use [47], and rates of word use are in turn subject to great fluctuations over time, as a reflex of shifting user behavior and shifting topics. For a lexical innovation to survive in the language, it must avoid an absorbing boundary near 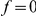, at which it is used so rarely that no one can learn it. Our investigation of the relationship between frequency change and dissemination change shows that a key to success beyond short-term fluctuations is increasing frequency (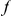) hand-in-hand with increasing dissemination (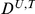). The success of the P-words in our case study can be understood by considering that exogenous forcing by external events allowed them to overcome the handicap of low dissemination values. S-words, selected to exemplify more endogenous dynamics, behaved more like words in general by displaying higher dissemination values when rising.

Word frequency affects word fate at historical time scales when different forms compete to express the same meaning [14], [15], [34]. Why did frequency not prove to be important in the dynamics of the whole vocabulary, as studied here? The language system has strong functional pressures for words to be distinct from each other, in both form and meaning [6], [41], [42], [45], [48]. Although dictionaries use words to explain the meanings of other words, and thesauri group together words with related meanings, true synonymy is very rare [49], [50]. For words which might seem to be synonyms, such as *soda* vs. *pop*, or *yes* vs. *yup*, there is normally a difference in dialect, formality, or other contextual factors governing the use of the word. Because almost every word is learned with a distinctive meaning (or set of meanings), and replication has low error rates, it follows that most words do not have a direct competitor for exactly the same meaning and contexts of use. If an active competition between two forms develops historically, then both can survive if they develop distinctive roles within the space of the lexical, syntactic, and pragmatic components of the linguistic system. For example, the English future auxiliary *gonna* is a new competitor for the older future *will*, but both survive because *gonna* is preferentially used in some constructions (such as questions), whereas *will* is preferentially used in others (such as the main clauses of conditionals) [51]. Reference [51] indeed uses the term *niche* to characterize these distinctive components in the usage of different future expressions, suggesting that differentiated niches are critical to their ongoing use in the language. These results complement those presented here by analyzing dimensions of the word niche that are internal to the linguistic system. The picture presents strong parallels to the exclusion principle in evolutionary biology, which states that occupying distinct niches protects species from competition [52]. Similar reasoning can also be applied to explore the competition between entire languages. In a model of language competition that assumes the speakers to be monolingual, distinct languages are similarly predicted to survive only if they are spoken by distinct, partially unmixed populations [53]. This prediction is attenuated if bilingualism in itself has high value or status as a human capability [54], permitting bilinguals to occupy a social position that is not available to monolinguals.

Diversity therefore depends on the diversity and viability of the individual niches. For biological species, the size of the geographical range and the species duration are correlated [18], [19]. In studies of the lexicon, the individual words assume the role of species, and we have shown that the relative extent of the word niche is associated with the likelihood of a favorable or unfavorable fate. But we have also shown that the relative extent of the word niche does not provide the whole story about viability. In population biology, exogenous events such as asteroid impacts can overcome the general statistical trends associated with dissemination. The same thing is true here, where exogenous events such as inventions and wars can overcome general statistical trends associated with the dissemination of words. This generalization is further illustrated by the recent finding that censorship can induce large and distinctive deviations from typical frequency trajectories for the names of people [34].

We found that 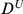 and 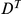 are positively correlated, but still provide distinct information. A positive correlation is expected because individuals have characteristic interests. Further mechanisms contributing towards this correlation result from the participation of individuals in social and geographical structures. For example, these can cause clumping in product use, as shown by profiling the Internet for software products [55], which entails clumping of the words used to discuss those products. Structures in the social network can even contribute directly to product adoption, because the usefulness of many products (such as high-tech innovations) can depend on the number of neighbors who already use the product [23], [56]. These same mechanisms pertain to other words, insofar as concepts and opinions resemble products.

We suggest, however, that other mechanisms limit the correlation between 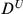 and 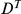, and explain the striking degree to which individuals were found to use different words in discussing the same topic. The variety in human social identities is thought to provide an impetus for innovation in modes of expression, as discussed in classic works of sociolinguistics [35], [36], [57]. Because people tend to associate with people like themselves, the variety in social identities can also give rise to clusters within social networks [58], and these clusters can in turn hinder lexical convergence [46], [57], [59]. The fundamental principles of discourse call for one to strike a balance between anchoring contributions in what the listener already knows, and providing novel and relevant information [60]. Online discourse can be viewed as a collective exploration of the conceptual world [61]. It follows from this study that the most engaging and fruitful discourse is discourse in which people cooperate in differentiating themselves and what they say.

## Methods

### 

#### Datasets

Usenet group archives are available at http://groups.google.com. The smallest unit of text is the *post*. Each post is attributed to a *user* and belongs to a *thread* (as defined by an initial post and all replies to it). We focus on two Usenet groups from their first post through 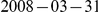: (i) comp.os.linux.misc, which concerns Linux operating systems, includes 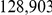 users and 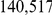 threads beginning 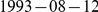; (ii) rec.music.hip-hop, which is devoted to hip-hop music, has 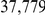 users and 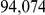 threads beginning 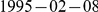. The activity of users in Usenet groups is bursty [32] and heterogeneous [13]. In the comp.os.linux.misc group, for example, the average user contributes 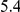 posts and remains active for 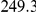 days, but the most persistent users have more than 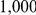 posts over more than 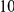 years. The average thread has 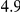 posts and is active for 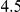 days, but the longest threads have more than 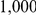 posts over 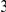 years. See Text S1 in Supporting Information S1 for information about preprocessing of the text, and Figures S1 and S3 in Supporting Information S1 for information about the fat-tailed distributions that characterize these groups.

#### Baseline model

The expected number of users 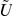 in Eq. (1) is calculated by assuming that all words are randomly shuffled, while holding constant the number of users and the number of words per user. Let 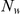 be the number of occurrences of the word 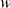, 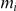 be the total number of words contributed by user 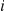, and 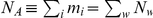. The probability that the 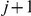 th occurrence of 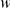 does not belong to user 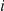 is given by 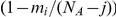. The probability 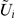 that user 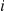 used word 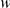 at least once is calculated as the complement of the probability of not using it:
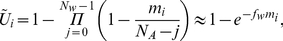
(3)where the approximation is valid for 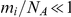 and 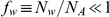. This corresponds to a Poissonian baseline model with a fixed probability of using 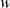 given by the observed word frequency 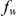. The error in the approximation is smaller than 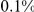 for the datasets we consider. This approximation was used in all calculations involving the untrimmed datasets, while the exact relation was used for the trimmed datasets. An analogous procedure is used for the calculation of the expected number of threads 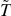.

## Supporting Information

Supporting Information S1 (PDF)

## References

[1] Onnela J-P, Saramäki J, Hyvönen J, Szabó G, Lazer D (2007). Structure and tie strengths in mobile communication networks.. Proc Natl Acad Sci USA.

[2] González MC, Hidalgo CA, Barabási A-L (2008). Understanding individual human mobility patterns.. Nature.

[3] Malmgren RD, Stouffer DB, Motter AE, Amaral LAN (2008). Poissonian explanation for heavy tails in e-mail communication.. Proc Natl Acad Sci USA.

[4] Seshadri M, Machiraju S, Sridharan A, Bolot J, Faloutsos C (2008). Mobile call graphs: Beyond power-law and lognormal distributions..

[5] Kuiper K (2006). Knowledge of language and phrasal vocabulary acquisition.. Behav Brain Sci.

[6] Davis MH, Gaskell MG (2009). A complementary systems account of word learning: Neural and behavioral evidence.. Phil Trans R Soc B.

[7] Munat J (2007). Lexical Creativity, Texts and Contexts.

[8] Kleinberg J (2003). Bursty and hierarchical structure in streams.. Data Min Knowl Dis.

[9] Cattuto C, Loreto V, Pietronero L (2007). Semiotic dynamics and collaborative tagging.. Proc Natl Acad Sci USA.

[10] Hotho A, Jäschke R, Schmitz C, Stumme G (2006). Information retrieval in folksonomies: Search and ranking.. Lec Notes Comput Sc.

[11] Neuman Y, Nave O, Dolev E (2011). Buzzwords on their way to a tipping-point: A view from the blogosphere.. Complexity.

[12] Crystal D (2006). Language and the Internet.

[13] Fisher D, Smith MA, Welser HT (2006). You are who you talk to: Detecting roles in Usenet Newsgroups.. Proc 39th Annual Hawaii Intl Conf Syst Sci.

[14] Pagel M, Atkinson A, Meade A (2007). Frequency of word-use predicts rates of lexical evolution throughout Indo-European history.. Nature.

[15] Lieberman E, Michel JB, Jackson J, Tang T, Nowak MA (2007). Quantifying the evolutionary dynamics of language.. Nature.

[16] Fontanari JF, Perlovsky LI (2004). Solvable null model for the distribution of word frequencies.. Phys Rev E.

[17] Colwell RK, Futuyama DJ (1971). On the measurement of niche breadth and overlap.. Ecology.

[18] Foote M, Crampton JS, Beu AG, Cooper RA (2008). On the bidirectional relationship between geographic range and taxonomic duration.. Paleobiology.

[19] Jablonski D (2005). Mass extinctions and macroevolution.. Paleobiology.

[20] Nettle D (1999). Is the rate of linguistic change constant?. Lingua.

[21] Wichmann S, Stauffer D, Schulze C, Holman EW (2008). Do language change rates depend on population size?. Adv Complex Syst.

[22] Lupyan G, Dale R (2010). Language structure is partially determined by social structure.. PLoS ONE.

[23] Watts D, Dodds P (2007). Inuentials, networks, and public opinion formation.. J Consum Res.

[24] Salganik ML, Dodds PS, Watts DJ (2005). Experimental study of inequality and unpredictability in an artificial cultural market.. Science.

[25] Eble C (1996). Slang and Sociability: In-group Language among College Students.

[26] Smitherman G (2000). Black Talk: Words and Phrases from the Hood to Amen Corner.

[27] Sornette D, Albeverio A, Jentsch V, Kantz H (2005). Endogenous versus Exogenous Origins of Crises.. Extreme Events in Nature and Society.

[28] Crane R, Sornette D (2008). Robust dynamic classes revealed by measuring the response function of a social system.. Proc Natl Acad Sci USA.

[29] Church KW, Gale WA (1995). Poisson mixtures.. Nat Lang Eng.

[30] Manning CD, Schütze H (1999). Foundations of statistical natural language processing.

[31] Serrano MA, Flammini A, Menczer F (2009). Modeling statistical properties of written text.. PLoS ONE.

[32] Altmann EG, Pierrehumbert JB, Motter AE (2009). Beyond word frequency: Bursts, lulls, and scaling in the temporal distributions of words.. PLoS ONE.

[33] Chesley P, Baayen RH (2010). Predicting new words from newer words, Lexical borrowings in French.. Linguistics.

[34] Michel J-B, Shen YK, Aiden AP, Veres A, Gray MK (2011). Quantitative analysis of culture using millions of digitized books.. Science.

[35] Milroy L (1980). Language and Social Networks.

[36] Eckert P (2000). Linguistic Variation as Social Practice.

[37] Lerman K, Hogg T (2010). Using a model of social dynamics to predict popularity of news..

[38] Szabo G, Huberman BA (2010). Predicting the popularity of online content.. Commun ACM.

[39] Fortunato S, Flammini A, Menczer F, Vespignani A (2006). Topical interests and the mitigation of search engine bias.. Proc Natl Acad Sci USA.

[40] Schifanella R, Barrat A, Cattuto C, Markines B, Menczer F (2010). Folks in folksonomies: Social link prediction from shared metadata..

[41] Steels L (1997). The synthetic modeling of language origins.. Evol Commun.

[42] Komarova NL, Nowak M (2001). The evolutionary dynamics of the lexical matrix.. Bull Math Biol.

[43] Dijksterhuis A, Bargh JA (2001). The perception-behavior expressway: Automatic effects of social perception on social behavior.. Adv Exp Soc Psychol.

[44] Nickerson DW (2008). Is voting contagious? Evidence from two field experiments.. Am Polit Sci Rev.

[45] Kirby S, Cornish H, Smith K (2008). Cumulative cultural evolution in the laboratory: An experimental approach to the origins of structure in human language.. Proc Natl Acad Sci USA.

[46] Hruschka DJ, Christiansen MH, Blythe RA, Croft W, Heggarty P (2009). Building social cognitive models of language change.. Trends Cogn Sci.

[47] Blythe RA, Smith K, Smith ADM (2010). Learning times for large lexicons through cross-situational learning.. Cogn Sci.

[48] Baronchelli A, Gong T, Puglisi A, Loreto V (2010). Modeling the emergence of universality in color naming patterns.. Proc Natl Acad Sci USA.

[49] Clark EV (1990). On the pragmatics of contrast.. J Child Lang.

[50] Wexler P, Culicover P (1980). Formal Principles of Language Acquisition.

[51] Torres Cacoullos R, Walker JA (2010). The present of the English future: Grammatical variation and collocations in discourse.. Language.

[52] Hardin G (1960). The competitive exclusion principle.. Science.

[53] Abrams DM, Strogatz SH (2003). Modeling the dynamics of language death.. Nature.

[54] Solé RV, Corominas-Murtra B, Fortuny J (2010). Diversity, competition, extinction: The ecophysics of language change.. J R Soc Interface.

[55] Trestian I, Kuzmanovic A, Ranjan S, Nucci A (2008). Unconstrained endpoint profiling (Googling the Internet)..

[56] Rogers EM (2003). Di_usion of Innovations.

[57] Labov W (2001). Principles of Linguistic Change: Social Factors.

[58] Wasserman S, Faust K (1994). Social Network Analysis.

[59] Lu Q, Korniss G, Szymanski BK (2009). The Naming Game in social networks: Community formation and consensus engineering.. J Econ Interact Coord.

[60] Grice HP, Cole P, Morgan JL (1975). Logic and Conversation.. Syntax and Semantics, Vol. 3: Speech Acts.

[61] Cattuto C, Barrat A, Baldassarri A, Schehr G (2009). Collective dynamics of social annotation.. Proc Natl Acad Sci USA.

[62] Kruskal W (1987). Relative importance by averaging over orders.. Am Stat.

