# Supplementary material for: Niche as a Determinant of Word Fate in Online Groups
Source: PLoS One. 2011 May 12;6(5):e19009. doi: 10.1371/journal.pone.0019009 (PMC3093376; doi:10.1371/journal.pone.0019009)
Supplement: Supporting Information S1 — (PDF) [file pone.0019009.s001.pdf]

Niche as a determinant of word fate in online groups
Eduardo G. Altmann, Janet B. Pierrehumbert, Adilson E. Motter

Supporting Information S1

Text S1. Filtering of data

In order to avoid spam present in the database, we eliminate all threads composed by a single post unless the author posted at least 5 times in threads with more than one post. Inside each post we consider only new inputs to the text, i.e., we omit parts of the text quoted from previous posts. Signature blocks (texts systematically placed at the end of posts by some users) were not removed because their content is deliberately chosen by the user. The use of signature blocks in Usenet is analogous to the use of formulaic expressions in other linguistic genres (e.g., greetings, farewells, sales transactions) that legitimately affect token frequency for constituent words.

Words are taken to be strings of characters separated from other strings by white space. In addition to space, tab, and newline, the character underscore (_) as well as all punctuation marks (. ! ? : ; ,) are treated as white spaces. However, apostrophe (') and hyphen (-) are not. This means that web and email addresses are broken up into their component parts, whereas expressions such as *weren't* and *e-mail* are treated as single words. Lines starting with *http://* are eliminated beforehand. Capitalization is removed, so that instances of the same word in sentence-initial and sentence-medial position are tabulated together. Strings consisting entirely or partly of non-alphanumeric characters other than $ and @ (e.g., #,%,&,*) are removed. No further lemmatization of purely alphabetic strings was imposed, with the result that all related words (e.g., singular and plural) are treated as distinct.

Text S2. Selection of target words

We are interested in words that first became popular during the lifetimes of the groups. Target words are selected that have negligible levels of use during the first 2.5 years of each group, and substantial use during the group's heyday. As shown in Figure 6CD (main text), in more recent times there is a clear reduction in the activity of the groups. Further analysis indicates that this reduction is accompanied by a concurrent deterioration in the informativeness of the postings. To avoid the selection of words used exclusively during this period, we require that at least 40% of target word uses occur prior to the time when the activity on the group fell to under a quarter of its peak level. This cutoff falls in 2005 for the rec.music.hip-hop group and 2007 for the comp.os.linux.misc group. In addition, we avoid the inclusion of words that are used predominately by single individuals, as we are interested in words that rose in the community more generally. Therefore, the following heuristics are adopted: no more than 20% of occurrences in a single month, no more than 40% of occurrences by a single user, and no more than 80% of occurrences by five users.

P-words are identified on a case-by-case basis among the most frequent words satisfying these criteria. S-words are identified with the help of dictionaries of Internet vocabulary and are selected from words with more than 100 appearances over the life time of the database. The following dictionaries of Usenet terms and Internet slang were used to identify words of interest: David Crystal's list of abbreviations, pp. 85-86 [1]; The Jargon File 4.3.3 [2]; the Wiktionary appendix on English Internet Slang [3]; and the Internet Slang Dictionary & Translator [4]. This leads to comparable counts for P-words and S-words in both groups, as shown in Tables S1-S4.

Text S3. Trimming scheme

For each half-year time window, the data are trimmed as follows:

1. *Standardize user contribution per thread*: Combine all posts of the same user in the same thread and define it as a single post for the purpose of the following analysis. Then rank the users from $i = 1$ to N and the threads from $j = 1$ to M, starting from the ones with the largest number of posts. Two auxiliary vectors store the number of posts, $\mathbf{u} = (u_i)$ and $\mathbf{t} = (t_j)$, where u_i is the number of posts of user i and t_j is the number of posts of thread j.

2. *Standardize the size of all posts*: Discard all posts smaller than the median in the window and randomly remove words from the others to redefine them to have exactly the same number of words, given by the median.

3. *Match number of users and threads*: Discard the set of $N-M$ users with smallest number of posts if $N > M$ and discard the set of $M-N$ threads with smallest number of posts if $N < M$, choosing randomly among those with equal number of posts. Repeat the process recursively after discarding any user and thread that is left with no posts,

and update vectors **u** and **t**. This step facilitates convergence even though the equality between the number of users and threads will generally be violated in the next step.

4. *Match the size of all threads and users*: For rank i, remove a random set of $u_i - t_i$ posts from user i if $u_i > t_i$ and a random set of $t_i - u_i$ posts from thread i if $u_i < t_i$. Starting from $i = 1$, proceed until all ranks have been considered.

5. *Match the low end of the distribution*: Apply steps 3 and 4 recursively to match the number of users and threads in the window under the additional constraint of having the exact same number of users and threads with a single post. To avoid depletion of the dataset, step 4 is in this case applied only to ranks in which the users or the threads have a single post.

Note that because the removals in step 4 are random to avoid sample biases, they also apply to posts from threads and/or users already treated and this will generally create a mismatch between the size of users and threads of same rank. As shown in Figures S2AB and S2DE for the half-year window centered on 1998-01-01, this mismatch is very small for both groups when compared to the original difference between the two distributions. Our definitions of word dissemination are not expected to be sensitive to such small differences. The only potential exception would be users and threads with single posts, since the words of such posts are not shuffled according to the baseline models used in the definition of $\hat{D}^T$ and $\hat{D}^U$, respectively. This potential artifact is eliminated by step 5, which assures the same number of threads and users with one post. Our procedure leads to statistically significant trimmed datasets, as exemplified in Figure S2CF, where we show the number of posts as well as the number of users and threads for the non-overlapping half-year time windows used in our analysis.

[1] Crystal D (2006) Language and the Internet. Cambridge: Cambridge Univ. Press.
[2] Chester County InterLink, Jargon File text Archive, http://jargon-file.org/archive/. Retrieved Jan. 29, 2009.
[3] Wiktionary, http://en.wiktionary.org/wiki/Appendix:Internet_slang. Retrieved Jan. 29, 2009.
[4] Internet Slang Dictionary & Translator, http://www.noslang.com/dictionary. Retrieved Jan. 29, 2009.

Table S1: **List of P-words in the comp.os.linux.misc group.** The total number of tokens N_w, users U_w, and threads T_w are calculated over the entire period, until 2008-03-31. The mean dissemination, $\langle D_w^{U,T} \rangle$, and standard deviation, $\sigma_{D_w^{U,T}}$, are calculated over all half-year windows that have $N_w > 5$, while $\langle D_w^{U,T} \rangle_\uparrow$ is the average calculated over the windows in the *rising period*. The rising period is defined as the period that starts with the first window for which $N_w > 5$ and lasts for the minimum between two years and half of the period to the window with the maximum frequency of the word. The centers of these windows are indicated by "Beginning" and "Peak", respectively, and the frequency in the peak window by f_{peak}.

Word	N_w	U_w	T_w	$\langle D_w^U \rangle_\uparrow$	$\langle D_w^U \rangle$	$\sigma_{D_w^U}$	$\langle D_w^T \rangle_\uparrow$	$\langle D_w^T \rangle$	$\sigma_{D_w^T}$	Beginning	$f_{\text{peak}} \times 10^6$	Peak
rpm	31910	6849	8439	0.63	0.51	0.08	0.51	0.40	0.08	1993-11-11	903.44	2002-04-07
kde	19236	5703	6726	0.54	0.66	0.10	0.32	0.49	0.11	1996-07-15	565.73	2001-11-05
suse	18776	5502	8057	0.78	0.70	0.08	0.75	0.61	0.09	1995-06-07	543.12	2004-08-13
gnome	11811	3853	4421	0.46	0.67	0.10	0.34	0.49	0.08	1997-07-28	399.05	2000-09-01
mandrake	11621	3870	5660	0.75	0.71	0.08	0.70	0.66	0.07	1998-08-11	535.98	2002-06-16
usb	10917	2312	2868	0.66	0.54	0.09	0.57	0.42	0.11	1996-10-21	698.41	2007-03-14
deja	8520	3202	5654	0.77	0.83	0.09	0.80	0.89	0.12	1996-04-15	913.20	2000-11-13
google	7230	2200	4069	0.89	0.81	0.10	0.89	0.81	0.11	1999-02-17	580.03	2006-06-07
ssh	7213	2083	2478	0.72	0.61	0.08	0.60	0.46	0.09	1995-11-25	337.65	2002-12-06
xp	5844	1964	2068	0.54	0.69	0.19	0.53	0.48	0.12	1994-06-30	445.49	2005-05-09
grub	5555	1238	1339	0.61	0.55	0.11	0.57	0.37	0.11	1996-10-28	436.48	2006-03-28
mozilla	5487	1614	2106	0.81	0.63	0.13	0.86	0.57	0.16	1995-05-18	354.18	2002-09-04
win98	5472	2513	2526	0.57	0.67	0.11	0.45	0.55	0.09	1997-06-18	275.55	2000-02-27
raid	5381	1448	1608	0.62	0.51	0.13	0.44	0.38	0.10	1994-02-21	223.19	2004-11-22
hash	5092	707	2735	0.56	0.39	0.20	0.79	0.68	0.13	1994-05-04	432.71	2004-05-09
gnupg	4988	260	2177	0.12	0.23	0.17	0.76	0.66	0.15	1998-10-07	701.43	2004-07-17
fedora	4818	1299	2248	0.46	0.69	0.13	0.41	0.64	0.13	2003-04-26	437.25	2004-12-18
fat32	4478	1883	1803	0.63	0.65	0.09	0.35	0.47	0.09	1996-05-10	137.98	1998-05-10
rpms	4440	2013	2586	0.64	0.68	0.10	0.63	0.64	0.10	1995-09-08	121.04	2001-01-23
glibc	4300	1659	1964	0.57	0.60	0.09	0.54	0.51	0.10	1994-06-12	151.22	1998-04-21
dvd	4297	1313	1417	0.53	0.57	0.09	0.32	0.39	0.08	1997-04-23	321.90	2006-10-11
staroffice	3997	1885	1652	0.65	0.70	0.10	0.43	0.49	0.11	1995-12-19	128.05	1999-12-01
sha1	3946	268	2272	0.15	0.22	0.13	0.79	0.77	0.13	1997-09-06	419.87	2004-05-09
ext3	3945	1107	1256	0.57	0.57	0.09	0.52	0.42	0.10	1999-07-31	309.17	2006-11-30
cdrecord	3909	953	1023	0.35	0.39	0.09	0.35	0.30	0.09	1997-01-28	185.59	2002-11-22
icq	3798	911	2149	0.41	0.36	0.13	0.68	0.70	0.14	1997-03-20	172.46	2001-04-06
vmware	3705	1076	1117	0.53	0.51	0.09	0.41	0.33	0.08	1998-12-19	150.95	2004-01-01
ubuntu	3411	854	1207	0.44	0.60	0.15	0.44	0.48	0.09	2004-07-13	524.59	2006-12-04
gimp	3405	1394	1320	0.51	0.61	0.11	0.44	0.46	0.11	1995-08-27	130.43	1998-05-09
pdf	3386	1137	1007	0.59	0.59	0.09	0.35	0.36	0.10	1995-06-18	168.45	2003-02-22
fetchmail	3321	975	799	0.54	0.46	0.17	0.39	0.30	0.13	1996-07-26	226.24	2003-10-05
dhcp	3270	1281	1224	0.52	0.59	0.07	0.35	0.42	0.08	1995-06-02	135.60	2002-06-17
mp3	3182	1286	1273	0.60	0.63	0.10	0.44	0.45	0.10	1996-10-11	173.92	2007-02-17
knoppix	3070	794	1188	0.64	0.61	0.11	0.45	0.51	0.12	2002-06-16	290.46	2004-12-23
mysql	2972	1128	977	0.60	0.59	0.09	0.41	0.39	0.07	1996-09-17	165.58	2005-03-21
gentoo	2631	643	1210	0.86	0.52	0.16	0.72	0.56	0.10	2001-11-13	188.13	2002-09-03
cnet	2627	1448	1969	0.64	0.78	0.22	0.61	0.79	0.20	1997-04-27	398.87	2000-04-14
nvidia	2481	851	881	0.70	0.59	0.11	0.56	0.44	0.11	1997-07-21	149.45	2007-04-11

Table S2: **List of S-words in the comp.os.linux.misc group.** The definitions are the same as in Table S1.

Word	N_w	U_w	T_w	$\langle D_w^U \rangle_\uparrow$	$\langle D_w^U \rangle$	$\sigma_{D_w^U}$	$\langle D_w^T \rangle_\uparrow$	$\langle D_w^T \rangle$	$\sigma_{D_w^T}$	Beginning	$f_{\text{peak}} \times 10^6$	Peak
distro	9796	2778	5016	0.63	0.65	0.07	0.70	0.68	0.08	1997-05-03	470.13	2006-05-13
hth	7117	1678	6474	0.52	0.46	0.10	1.04	1.08	0.05	1994-12-15	230.51	2007-04-01
distros	4417	1491	2645	0.67	0.68	0.08	0.77	0.72	0.06	1997-11-17	252.92	2006-11-25
iirc	3481	1198	2819	0.73	0.64	0.11	0.89	0.91	0.05	1995-06-05	113.62	2004-11-09
troll	3140	1232	1188	0.64	0.70	0.14	0.46	0.48	0.12	1994-02-15	270.72	2005-06-20
spammers	2404	570	1155	0.41	0.46	0.20	0.71	0.59	0.17	1996-09-29	185.52	2003-07-03
lol	961	518	685	0.89	0.78	0.14	0.86	0.79	0.11	1997-07-25	59.14	2006-11-16
y2k	910	326	332	0.56	0.51	0.17	0.31	0.42	0.17	1996-10-03	59.45	1999-10-24
plonk	634	264	420	0.70	0.70	0.19	0.71	0.73	0.15	1997-07-24	62.17	2005-08-13
boxen	580	264	460	0.81	0.69	0.20	0.93	0.84	0.10	1994-11-08	42.37	2001-04-24
wtf	383	249	310	0.85	0.86	0.15	0.69	0.87	0.14	1995-06-23	30.42	2006-04-11
eula	378	154	164	0.46	0.61	0.20	0.28	0.49	0.25	1997-07-02	39.68	2005-07-10
bsod	278	167	132	0.76	0.77	0.20	0.49	0.58	0.25	1997-08-08	19.36	2002-07-29
istr	275	128	253	0.76	0.70	0.19	0.91	0.94	0.09	1996-12-20	20.11	2004-12-25
blog	227	86	139	0.34	0.49	0.16	0.43	0.63	0.20	2003-11-15	31.30	2006-08-29
addy	126	84	99	0.78	0.74	0.19	0.87	0.77	0.17	1998-04-07	7.19	2004-05-02
ianal	113	80	69	0.84	0.82	0.17	0.48	0.54	0.17	1998-01-09	11.68	2004-12-24

Table S3: List of P-words in the rec.music.hip-hop group. The definitions are the same as in Table S1.

Word	N_w	U_w	T_w	$\langle D_w^U \rangle_\uparrow$	$\langle D_w^U \rangle$	$\sigma_{D_w^U}$	$\langle D_w^T \rangle_\uparrow$	$\langle D_w^T \rangle$	$\sigma_{D_w^T}$	Beginning	$f_{\text{peak}} \times 10^6$	Peak
rmhh	19790	1308	8823	0.64	0.51	0.14	0.78	0.75	0.09	1995-05-10	2500.78	2005-05-08
eminem	12363	2061	4439	0.36	0.79	0.15	0.59	0.55	0.07	1997-10-16	629.58	1999-01-08
mos	10405	1430	4457	0.56	0.69	0.18	0.58	0.64	0.10	1996-04-10	844.10	1999-12-24
bush	8031	1101	2465	0.85	0.66	0.16	0.79	0.56	0.18	1995-05-10	118.70	2004-10-23
kweli	3737	842	1735	0.42	0.70	0.13	0.57	0.59	0.11	1997-09-07	232.48	1998-11-18
iraq	3428	519	853	0.53	0.53	0.15	0.59	0.41	0.20	1995-12-19	422.75	2004-12-08
doom	3096	560	1605	0.88	0.61	0.17	0.85	0.61	0.13	1995-05-10	200.82	2005-12-18
cent	2775	637	1641	0.86	0.77	0.15	0.82	0.77	0.14	1995-05-10	564.05	2005-09-07
pun	2707	970	1566	0.97	0.83	0.11	0.96	0.73	0.16	1995-05-10	153.58	1998-07-17
peas	2675	452	1664	0.96	0.59	0.25	1.01	0.76	0.11	1995-07-02	362.22	2000-08-03
dvd	2450	579	1128	0.41	0.62	0.16	0.43	0.53	0.11	1997-08-05	178.87	2006-05-13
icq	2302	257	1586	0.42	0.38	0.23	0.89	0.70	0.23	1997-08-08	197.85	1998-11-02
ja	2266	776	1242	0.84	0.79	0.13	0.77	0.67	0.12	1995-05-10	177.05	2002-10-16
talib	2225	700	1259	0.48	0.75	0.15	0.61	0.66	0.09	1997-09-05	111.60	1999-04-23
kanye	2199	363	863	0.62	0.66	0.08	0.57	0.54	0.09	2001-06-23	353.47	2004-04-24
dilated	1916	519	1117	0.47	0.68	0.15	0.82	0.68	0.14	1997-05-12	152.49	2000-09-09
slug	1813	335	842	0.40	0.54	0.16	1.01	0.62	0.23	1995-06-02	173.65	1999-11-26
google	1787	546	1256	0.84	0.90	0.13	0.79	0.82	0.06	2000-05-05	275.06	2005-10-29
jigga	1702	486	915	0.81	0.62	0.19	0.74	0.63	0.13	1997-09-30	139.23	2006-12-09
riaa	1346	304	649	0.60	0.62	0.24	0.38	0.53	0.23	1997-12-10	127.27	1998-11-11
kobe	1234	346	370	0.67	0.63	0.17	0.37	0.43	0.22	1996-11-10	96.82	2000-04-09
neptunes	1218	371	580	0.82	0.75	0.13	0.68	0.60	0.14	1999-04-28	127.97	2002-09-09
necro	1193	360	596	0.50	0.62	0.12	0.50	0.55	0.13	1996-11-21	60.96	2000-11-03
lif	1146	220	574	0.52	0.53	0.18	0.73	0.64	0.18	1997-01-18	155.86	2002-06-24
anticon	1129	324	457	0.49	0.64	0.12	0.59	0.48	0.14	1998-08-08	91.95	2000-05-09
blackstar	1081	378	675	0.63	0.79	0.13	0.71	0.72	0.15	1997-12-05	116.90	1998-12-11
blueprint	1080	368	577	0.84	0.75	0.14	0.76	0.68	0.16	1995-05-10	135.10	2001-11-30
saddam	1005	243	278	0.81	0.51	0.18	0.63	0.39	0.17	1996-01-29	181.55	2003-01-25

Table S4: **List of S-words in the rec.music.hip-hop group.** The definitions are the same as in Table S1.

Word	N_w	U_w	T_w	$\langle D_w^U \rangle_\uparrow$	$\langle D_w^U \rangle$	$\sigma_{D_w^U}$	$\langle D_w^T \rangle_\uparrow$	$\langle D_w^T \rangle$	$\sigma_{D_w^T}$	Beginning	$f_{\text{peak}} \times 10^6$	Peak
lol	8196	1452	5112	0.72	0.68	0.09	0.86	0.84	0.07	1996-05-31	506.44	2005-10-26
ot	2692	532	1655	0.88	0.62	0.18	0.95	0.79	0.15	1995-06-27	167.81	2000-09-29
troll	1766	597	883	0.82	0.72	0.18	0.64	0.59	0.12	1995-12-25	123.23	2006-01-06
wtf	1544	577	1252	0.65	0.75	0.11	0.83	0.87	0.10	1996-10-15	74.42	2005-10-11
chicks	1406	524	934	0.88	0.79	0.12	0.87	0.76	0.11	1995-05-10	71.91	2004-02-09
prolly	1115	281	943	0.82	0.57	0.18	0.92	0.90	0.07	1995-05-10	54.27	2000-08-07
copped	1013	344	836	0.83	0.67	0.15	0.96	0.87	0.07	1996-09-17	79.43	2006-05-08
bling	780	279	358	0.52	0.66	0.14	0.54	0.57	0.16	1999-03-24	62.76	2000-12-19
arse	765	202	623	0.67	0.59	0.24	0.93	0.88	0.10	1997-03-08	75.37	1999-10-03
ps2	564	172	157	0.60	0.58	0.14	0.24	0.35	0.17	1999-06-18	60.97	2002-01-03
trolls	473	232	325	0.88	0.79	0.16	0.85	0.73	0.12	1997-08-13	41.90	2004-04-27
otp	402	180	280	0.60	0.71	0.15	0.65	0.74	0.12	1998-01-13	28.60	1999-12-11
iirc	397	132	363	0.56	0.62	0.17	0.94	0.95	0.06	1999-02-06	55.81	2006-06-05
congrats	332	196	234	0.92	0.91	0.14	0.95	0.76	0.18	1998-02-01	49.09	2006-07-31
lmao	330	155	289	0.69	0.70	0.17	0.90	0.90	0.09	1998-02-23	21.08	2003-11-18
twat	278	131	226	0.62	0.71	0.18	0.94	0.85	0.15	1997-03-31	55.37	2006-10-16
arsed	224	91	207	0.53	0.67	0.18	0.94	0.93	0.10	1998-04-21	24.48	2006-12-31
innit	212	85	195	0.49	0.63	0.15	0.94	0.95	0.05	1998-03-05	33.13	2005-12-25
addy	202	135	167	0.79	0.81	0.13	0.90	0.83	0.12	1998-04-28	15.32	2005-01-22
omg	194	66	145	0.40	0.55	0.25	0.88	0.82	0.22	1999-12-17	34.59	2005-09-27
lurker	178	138	149	0.93	0.92	0.09	0.82	0.82	0.14	1997-10-15	8.84	2005-05-21
kfc	142	87	82	0.56	0.70	0.21	0.49	0.60	0.24	1998-03-21	11.67	2003-10-12
plonk	135	90	94	0.73	0.80	0.17	0.57	0.71	0.18	2001-08-04	23.19	2005-07-08
afaik	131	79	123	0.81	0.77	0.18	0.90	0.93	0.09	2000-01-05	17.04	2006-05-31
roflmao	108	60	96	0.59	0.64	0.15	0.91	0.85	0.20	1998-01-07	6.38	2000-08-20
yuo	107	45	85	0.88	0.43	0.24	0.86	0.81	0.26	1998-01-20	13.45	2005-02-15
snes	106	56	57	0.64	0.64	0.21	0.46	0.48	0.13	1998-04-07	12.19	2001-08-25
wmd	104	42	45	0.57	0.51	0.20	0.48	0.45	0.21	2002-10-30	16.56	2005-08-04
wank	104	61	78	0.49	0.69	0.20	0.61	0.74	0.18	1998-09-14	14.62	2002-07-24
rotflmao	100	56	85	0.81	0.69	0.24	0.83	0.82	0.14	1998-03-02	12.47	2004-07-01

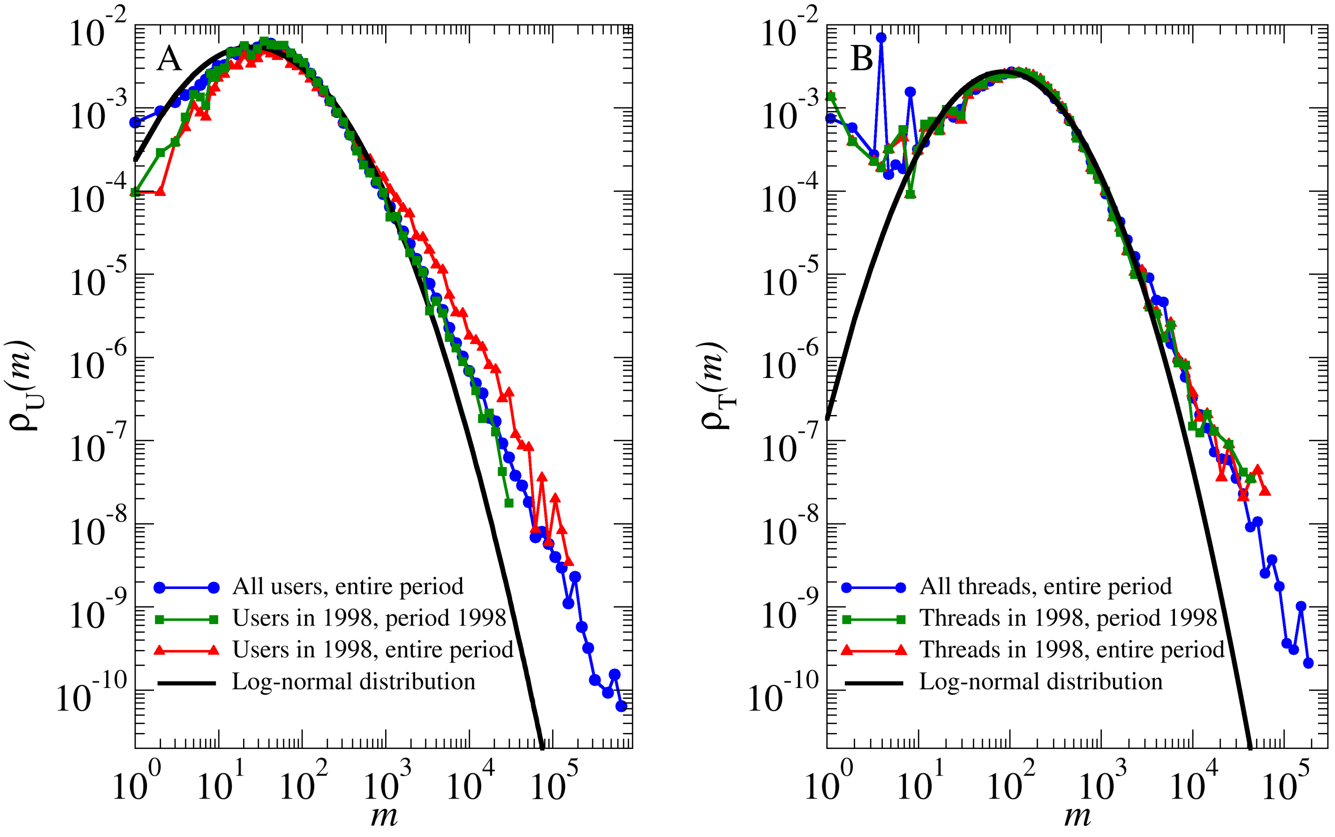

Figure S1: **Word distributions per user and per thread in the comp.os.linux.misc group. A**, Probability density function of users that contributed m words to the text. Blue: all users in the entire database. Green: users active in the half-year window centered on 1998-01-01, words in posts during the same half-year window. Red: users active in the half-year window centered on 1998-01-01, words in posts for these users over the lifetime of the database. Black: log-normal distribution obtained by fitting the two first moments of the green distribution. **B**, The same as in panel **A** for threads. Logarithmic bin sizes used in all cases. Similar results are found for the rec.music.hip-hop group.

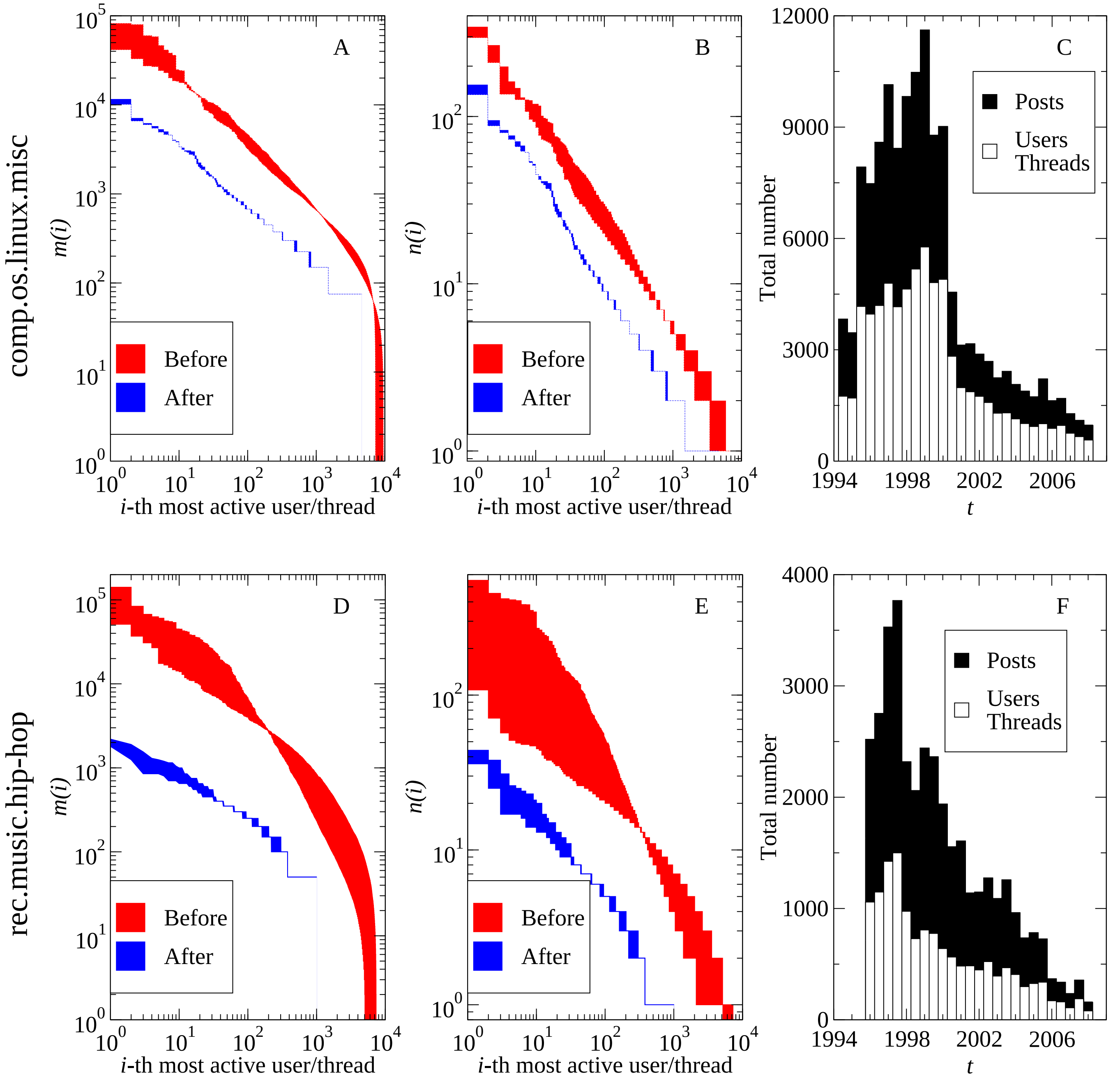

Figure S2: **Distributions for the trimmed datasets of the comp.os.linux.misc and rec.music.hip-hop groups.** **A**,**D**, Difference between the number m of words of the i-th most active user and thread before and after trimming for a typical half-year window, centered on 1998-01-01. **B**,**E**, Difference between the number n of posts of the i-th most active user and the i-th most active thread, calculated both before and after trimming for the window centered on 1998-01-01. **C**,**F**, Number of posts and number of users in the trimmed datasets for each non-overlapping half-year window centered at t. The number of threads is equal to the number of users in the trimmed datasets.

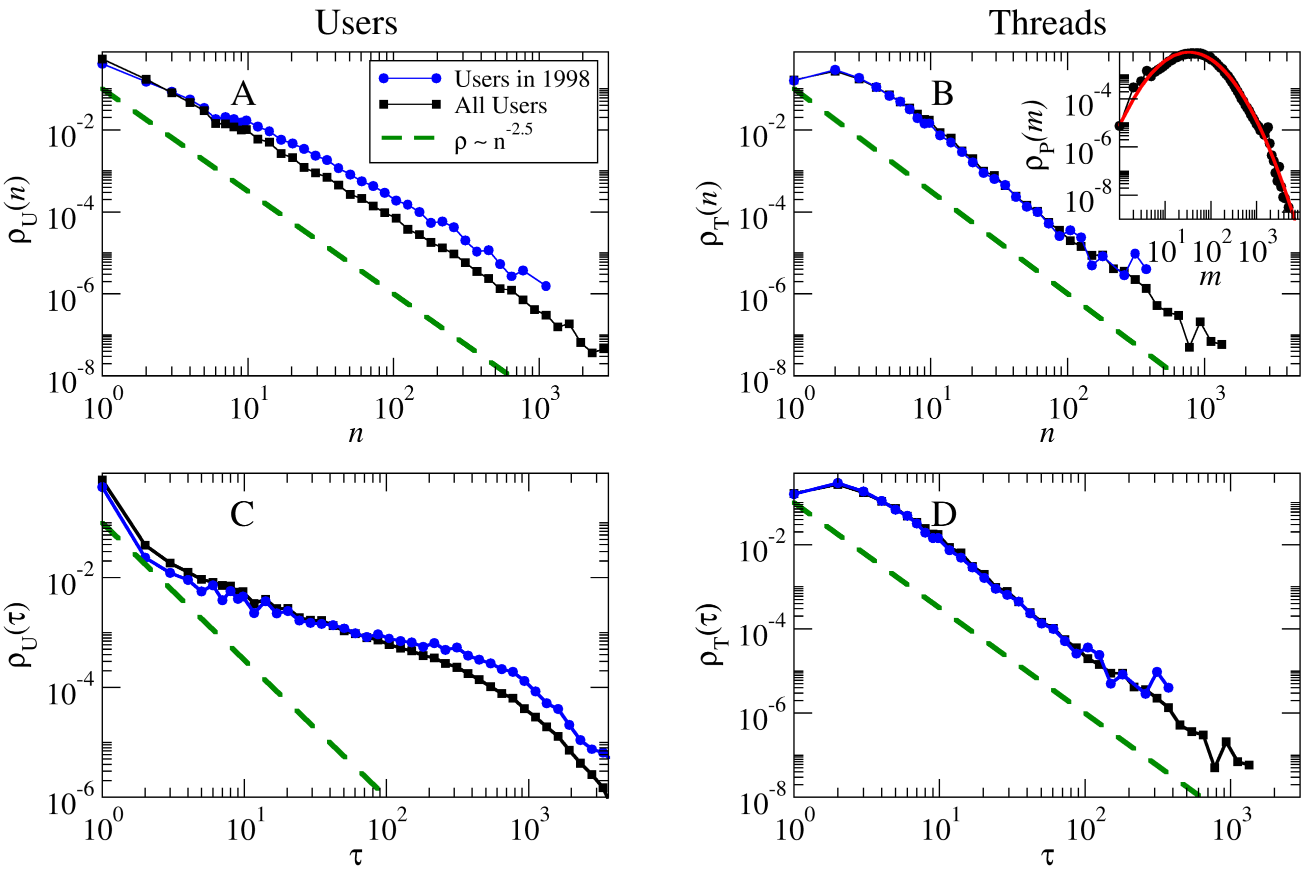

Figure S3: **Analysis of user and thread activity in the comp.os.linux.misc group. A**,**B**, Distribution of users with n posts (**A**) and of threads with n posts (**B**). Inset: distribution of the number of words per post with a log-normal fit (red line). **C**,**D**, Distribution of users with activity interval τ (**C**) and of threads with activity interval τ (**D**), where the activity interval is expressed as the number of days between the first and the last post. Blue lines: activity over the entire database of users and threads active in the half-year window centered on 1998-01-01. Black lines: activity of all users and threads in the database. Green dashed lines: power law with an exponent $\alpha = -2.5$, provided as a visual reference for the tails of the distributions. In panels **A**,**C**, the difference between the tails of the blue and black curves arises because the set of users active in the 1998-01-01 window, which is early in the lifetime of the group, have more opportunity to contribute a large number of posts than users joining the discussion group later. Logarithmic binning is used in all cases. Similar results are found for the rec.music.hip-hop group.
